# Implementing youth participatory action research at a continuation high school

**DOI:** 10.1111/1475-6773.14190

**Published:** 2023-06-06

**Authors:** Natalie A. Lárez, Jill D. Sharkey, Shannon Frattaroli, Elena Avila, Andres Medina

**Affiliations:** ^1^ Department of Counseling, Clinical, and School Psychology University of California Santa Barbara California USA; ^2^ Health Policy Research Scholars Program National Program Center The Johns Hopkins Bloomberg School of Public Health Baltimore Maryland USA; ^3^ Youth Student Researchers from the Continuation High School Santa Barbara California USA

**Keywords:** child and adolescent health, mental health, mental health promotion, social determinants of health, survey research and questionnaire design

## Abstract

**Objective:**

To describe the process of implementing a Youth Participatory Action Research (YPAR) project at a continuation high school (CHS) and share the results of a youth‐designed research project that explores barriers to high school completion.

**Data Sources and Study Setting:**

YPAR was implemented across three cohorts at a CHS in the central coast of California from 2019 to 2022. Student survey respondents were enrolled CHS students between March and April 2021.

**Study Design:**

A modified YPAR curriculum integrating research methodology and social justice topics was used to guide student‐led research that resulted in a cross‐sectional survey.

**Data Collection:**

Field notes maintained by the first author documented the process of implementing YPAR including the curriculum, conversations, and research decisions and procedures. A student‐designed survey disseminated to all enrolled students resulted in 76 (66%) participant responses. The survey included 18 close‐ended questions and three narrative responses.

**Principal Findings:**

This study details how YPAR methodologies can be translated to a high school credit recovery program. For example, student cohorts were needed to maintain continuity over time. A student‐designed survey revealed that 72% of student respondents reported taking care of family members and illuminated high rates of depression symptoms.

**Conclusions:**

This study offers a detailed description of how we implemented YPAR at a credit recovery program and provides student‐driven perspectives on educational reform and evaluation. This project addresses the implementation and challenges of using YPAR to engage youth in transformational resistance to rapidly study and improve CHS' policy and practice.

**What is known on this topic**

- The majority of Youth Participatory Action Research (YPAR) (*n* = 44, 64.7%) has been published after 2009, indicating that YPAR research is still emerging.^19^
- Although YPAR is conducted mostly in school settings, there is limited research outlining the process of YPAR within continuation high schools (CHSs).
- YPAR implementation has demonstrated positive outcomes for participating youth, such as increased agency, improved interpersonal relationships, and improved academics.^19^

**What this study adds**

- We document the process of implementing YPAR within continuation and credit‐recovery programs across multiple cohorts of students with highly mobile youth.
- This study suggests that youth's family responsibilities and emotional distress may limit attendance, and completion, for CHS students.
- The present study highlights the benefit of using YPAR to solicit youth input especially in underserved student populations, such as CHS students.

## 1 INTRODUCTION

A continuation high school (CHS) is defined as an alternative high school diploma program tailored to support students who are 16 years or older, have not graduated, and are at risk of not graduating. CHS have fulfilled multiple roles, including citizenship training, vocational advising, and preventing dropout.^1^ Students who attend CHS face challenges that their traditional high school peers may not, such as potential involvement with the foster care system and lower socioeconomic status.^2^ These challenges may affect school completion and graduation rates.

California is the most diverse state in terms of socioeconomics, household composition, and cultural identity.^3^ Among the State's K–12 public school students, about 54.9% identify as Hispanic or Latiné, 22.4% identify as White, 9.3% identify as Asian, and 5.3% identify as African American.^4^ About 145,000 students (2.3%) attend some form of public alternative education program (CDE),^5^ which enroll higher proportions of youth of color, foster care youth, and/or youth from lower socioeconomic statuses (SES).^6^, ^7^ CHS make up the largest group of alternative education programs, with 425 schools supporting 50,000–80,000 students in the 2019–2020 school year.^5^ Given the particular needs of CHS students, equipping CHS to provide the resources and services to holistically support youth is critical.

CHS are responsive to California's compulsory education laws that require that students between the ages of 6 and 18 years to attend public school, with the exception of students enrolled in private education. CHS welcome students who are at least 16 and “at‐risk” of not graduating.^5^ CHS programs have been criticized as ‘dumping grounds’ for students who are not meeting milestones.^8^, ^9^ However, they have the potential to address a need by providing a supportive environment for students who have been failed by the traditional education system.

CHS are governed by national and state legislation that centers accountability by measuring student or teacher success and identifying low‐performing schools. For example, the Every Student Succeeds Act (ESSA)^10^ requires that state educational agencies to collaborate with stakeholders to develop and implement a plan to improve student outcomes under a Comprehensive Support and Improvement (CSI) plan in schools that fail to meet specified criteria for two consecutive years. CHS programs are more likely than comprehensive high schools to be identified for CSI because students who enter CHS are not “on‐track” to graduate, due to a lack of credit completion or chronic absenteeism. State educational agencies, including CDE, can also identify model CHS that demonstrate outstanding and engaging programs with comprehensive services.

### 1.1 School engagement and disengagement through a critical race theory lens

Critical Race Theory (CRT), which has been a cornerstone of education activism and reform since the turn of the Century, posits that race, ethnicity, and social class affect school experiences and performance for youth of color.^11^ Utilizing a CRT framework agitates the traditionally held belief that educational systems are impartial, color‐blind, race neutral, and provide equal opportunities to all students.^12^ For example, the link between student engagement and academic success is well‐established in the literature. Finn and Rock^13^ underscored that engagement in school is often a critical component of programs for students identified as “at‐risk.” Newman^14^ referred to student engagement as central to student success and called on school personnel to attend to engagement as a dropout prevention strategy.

CRT also provides a framework for understanding disengagement from the student perspective. Solorzano and Delgado Bernal^12^ discuss disengagement as school resistance and name four ways that marginalized youth resist schooling by expanding on Fine's^15^ initial dichotomous conceptualization: Self‐defeating behaviors, reactionary behaviors, conformist resistance, and transformational resistance, with transformational resistance offering the greatest possibility for social change.^12^ By viewing disengagement through a CRT and resistance framework, school systems can acknowledge that youth of color resisting can motivate individual and societal‐level change, and positive responses from schools can lead to transformational results.

CHS are in a unique position to serve Black and Latiné youth because these students are overrepresented in their ranks and may have non‐traditional academic needs resulting from high residential mobility, lower socio‐economic status, and foster care system involvement.^16^ Here again, CRT is helpful in situating these responsibilities within a larger system of oppression that has systematically disadvantaged Black and Brown communities. Research is needed to explore strategies that engage and promote the involvement of continuation school students in transformative resistance to ultimately support positive systems change that will help continuation schools to better serve historically marginalized students.

### 1.2 Youth Participatory Action Research

Youth Participatory Action Research (YPAR) is a youth engaged iteration of Participatory Action Research (PAR), includes the same values as PAR, and can be used to address inequities in education and support positive youth development.^17^ Rodriguez and Brown^18^ operationalize YPAR through three key principles: YPAR is grounded in youths' lived experiences and centers them as experts; YPAR is participatory—youth select research methods and guide the implementation process; and YPAR is transformative in that it shares a process with youth to challenge knowledge creation and realize system change.

YPAR includes youth researchers in every phase of research,^19^, ^20^ and emphasizes students' agency to enact change within their settings.^21^ Through YPAR, youth can examine problems and propose solutions in a way that recognizes their capabilities and acknowledges existing inequities. Research supports the transformational potential of this process. A systematic review of 63 YPAR projects across various settings in the US highlighted the most common positive outcomes for participating youth, which included increased levels of agency, leadership, academics, social competency, critical consciousness, interpersonal relationships, and an increased understanding of social justice issues.^22^ Thus, YPAR has been proven to positively impact systems while also improving the lives of youth participants.

### 1.3 YPAR in continuation schools

One systematic analysis reporting on YPAR research in schools identified 38 studies with a majority (71%) of studies being conducted within urban school settings, 21% in suburban settings, and the remainder (8%) in multiple school‐based settings or rural settings.^23^ Ozer^20^ noted that school policies (e.g., standardized testing, allocation of time for core standards, class scheduling, graduation requirements) are potential barriers to youth truly leading a YPAR project. Few (*n* = 2) YPAR studies involved continuation or alternative high schools.^23^ Notably, new research has been published with examples of YPAR being implemented within the CHS,^6^, ^24^ though examples are still limited. A gap in the literature exists that pays specific attention to YPAR implementation in continuation schools and addresses the unique needs of students, such as high mobility between various school settings. The power‐sharing principles of YPAR, where youth are situated as partners, has been highlighted as a key element to strengthening the validity for youth from minoritized backgrounds.^20^

### 1.4 Study purpose

To address the shortcomings in the literature and in practice, we undertook a study to detail the process of implementing YPAR across three cohorts of youth co‐researchers within a CHS in California. We used YPAR to uncover youth perspectives about school reform practices in the context of low attendance and graduation rates. Similar to Solorzano and Delgado Bernal,^11^ we employed a CRT lens to challenge the common deficit discourse associated with youth in credit‐recovery programs and acknowledge the systemic oppression entrenched in the US education system. This paper aims to describe how the YPAR was implemented in a CHS and shares the outcomes of the project.

## 2 METHODS

### 2.1 Study design and setting

The school district initiated the university—school research collaboration. The participating CHS was eligible for CSI because its graduation rates were below the state target. The CHS in 2020 had an enrollment of 113 students of whom 93% were Latiné, 90.3% were classified as “socioeconomically disadvantaged,” and 19.5% were classified as “English Learners.”^23^ Youth at the participating high school were overrepresented in categories historically marginalized by the US education system relative to the district and county (Table 1). University researchers conducted a needs assessment, examined student strengths and needs, and implemented YPAR to identify root causes of low graduation rates. Teachers, school staff, university graduate and undergraduate students, and high school co‐researchers participated.

**TABLE 1:** Research context: race/ethnic demographics.

Race/ethnicity	% Latiné/Hispanic	% Black/African American	% Asian/Asian American	% Non‐Hispanic White
Local county	70.5	0.9	1.6	22.1
Local school district	59.9	0.8	2.7	32.6
High school	86.7	3.3	‐	8.9

### 2.2 Research team

The research team included members of the author team: a graduate student researcher (NL), three youth co‐researchers (EPA, JM, and AM) and a faculty research supervisor (JS). Decision‐making was shared among the graduate student researcher and the co‐researchers. The graduate student researcher is a first‐generation college graduate, Latina, and from a low‐resourced community; she is a doctoral student in school psychology and has received mental health training under the supervision of licensed school and clinical psychologists. The faculty research supervisor is trained as a school psychologist and conducts research on systems change to support vulnerable populations; she has a longstanding partnership with the school district and was the principal investigator for the research.

Since youth at the continuation of high school are highly mobile, it was common for the number of youth co‐researchers to fluctuate throughout the year. Youth co‐researchers were first identified by school staff and provided with information about days and times of the regular YPAR sessions. Following the initial staff recommendation, youth who were interested attended a YPAR session or information session prior to deciding if they wanted to continue as a YPAR member. Demographics of youth co‐researchers were not collected. Instead, conversations about immigration status, families, and connections to geographic location were discussed as a way to center youths' conceptions of their identities.

### 2.3 YPAR implementation

Through the YPAR process between Spring 2020 and Fall 2022, student co‐researchers developed and implemented a mixed methods approach to identify and describe the underlying reasons for low school attendance to understand factors that may contribute to low graduation rates. In response to the challenge conducting YPAR with a highly mobile student population (students are typically at the school between 3 months and 2 years), three groups of youth co‐researchers (Cohorts 1–3) participated. Each cohort was in place for 3–10 months, with an overlap for a few members to ensure continuity. Notably, the three cohorts of YPAR implementation were all affected by the COVID‐19 school closures and distance learning. The graduate student researcher adapted curriculum implementation and YPAR sessions to online Zoom sessions as necessary and awaited school approval for in‐person implementation when available.

The graduate student researcher adapted two YPAR curricula^25^, ^26^ to support implementation. Specifically, each outlined lesson focused on research methods (e.g., interviews, focus groups, photo voice, surveys) and social justice topics (e.g., culture, identity, stereotyping, and gender roles). Decisions about which curricula lessons to include were based on each cohort's research task. For example, in Cohort 1, the intention was to first equip youth co‐researchers with various research methodologies so they could select research methods. The graduate student researcher facilitated conversations and lesson plans twice a week throughout implementation. Conversations covered a range of topics, such as grant management and IRB processes, included opportunities for skill building and familiarizing the youth co‐researchers with research, and were responsive to youth identified interests. Youth co‐researchers developed their own goals for the project related to graduation, attendance, and barriers to high school completion, and they participated in selecting the implementation and data collection methods.

Since this YPAR project was carried out across three cohorts, Cohorts 2 and 3 were tasked to carry out Cohort 1's selected research methodology and to expand implementation. Two students from Cohort 1 returned for Cohort 2 and shared with Cohort 2 their experience in YPAR and the survey results. Cohort 2 also decided which survey variables to focus on based on their interest and frequency among student respondents. Cohort 2 developed focus group questions intended to explore student mental health and its effects on attendance and high school completion and began discussing possible methods for implementation in year 3. The graduate student researcher and a continuing co‐researcher from Cohort 2 shared the project with Cohort 3. Cohort 3 prepared to implement the focus groups and continued to workshop the focus group questions designed by Cohort 2.

During Cohort 3, the school was transitioning back to in‐person classes after COVID‐19 school closures, and they did not implement the focus groups because of the school's focus on crisis response and more urgent student supports during this time. The graduate student researcher adjusted and supported the school and the students through other avenues (e.g., drafting policy memos, curriculum consultation, state and district level data communication, training peer mentors). In addition, the sustainability of YPAR curricula, with modifications to support use by CHS students, was planned with school administration for a time when they would have the bandwidth to offer teacher‐led YPAR embedded in the leadership curriculum.

### 2.4 Student‐designed survey development

Through conversations facilitated and documented by the graduate student researcher, youth co‐researchers in Cohort 1 decided to develop a survey asking about mental health (anxiety and depression), substance use, family responsibility (e.g., taking care of younger siblings), safety, and transportation. In addition, some questions were specifically tailored to the students' experiences during COVID‐19 because the survey was fielded as students were returning to in‐person classes after attending school virtually for a year due to COVID‐19 school closures.

As co‐researchers were interested in exploring mental health symptom presentation for anxiety and depression, the graduate student researcher engaged with the co‐researchers about existing validated mental health questionnaires while also weighing student co‐researchers' priority of keeping the survey under 20 questions. The YPAR team decided to incorporate the Patient Health Questionnaire‐2 (PHQ‐2) and the Generalized Anxiety Disorder 2‐item (GAD‐2)^27^ screeners for depression and anxiety, respectively. The PHQ‐2 and GAD‐2 are designed to be self‐report screeners for symptoms and are not intended to be used as diagnostic tools. They have been validated as screeners in youth and demonstrate high sensitivity and specificity.^28^, ^29^ Other questions, such as those related to substance use, were modified from existing questions that were included in the California Healthy Kids Survey (CHKS). The CHKS is an annual survey that is administered to students from 7th‐12th grade in all California public schools. Youth co‐researchers shortened the question based on their previous experience taking the CHKS. For example, youth co‐researchers removed substance questions related to cigarette and vape use because they believed it was important to instead highlight alcohol, marijuana, and pill use. The final survey is included in Appendix S1.

### 2.5 Student‐designed survey data collection and analysis

The student‐designed survey was administered between March 31 and April 9, 2021. All student survey participants were recruited from the participating CHS. A total of 108 students, which was the total enrollment at the time, were invited to complete or opt out of the survey. Parents were notified and provided with a copy of the survey via the online parent portal. Parents had the opportunity to opt for their student out of participating. Demographic variables were not collected to maintain anonymity. The survey was disseminated through the web‐based survey platform Google forms. Data were downloaded to Excel and uploaded into SPSS Version 27 for descriptive analysis.

### 2.6 Field notes

Field notes maintained by the first author documented the process of implementing YPAR including the curriculum, conversations, and research decisions and procedures. The first author was an active participant in the YPAR process and completed field notes during and after YPAR sessions with youth co‐researchers to provide contextual information for informal conversations, research decisions, suggested adjustments from youth co‐researchers, and the general process of YPAR. Field notes were not formally analyzed but were used to inform the iterative process of participatory research and gather quotes on informal conversations with youth co‐researchers.

### 2.7 IRB statement

The University of California, Santa Barbara institutional review board (IRB) reviewed and approved this study.

## 3 RESULTS

### 3.1 YPARprocess

Table 2 presents specific details related to recruitment, method of participation, incentives, curriculum utilized, research tasks, and total number of youth co‐researchers across the three cohorts of YPAR implementation. In February 2020, 12 youth attended an initial interest meeting for the first cohort. Six committed to continuing as co‐researchers until the end of the school year, including the transition to virtual meetings over Zoom. Two co‐researchers graduated in March 2020. From February through June 2020, the remaining four students attended, on average, 70% of the 35 semi‐weekly Zoom sessions. Two co‐researchers from cohort one continued through March 2021, when recruitment for cohort two began. Recruitment was paused as the school transitioned back to in‐person schooling, and resumed once complete in April 2021. Four of the seven students who attended the interest meeting in April 2021 committed to attending YPAR sessions until May 2021. Two co‐researchers from cohort two continued into the next school year when cohort 3 began. In August 2021, five of eight new students identified by teachers decided to be a part of the third YPAR cohort. Youth co‐researchers intended to implement focus groups in November 2021, but YPAR plans were postponed because of administrative changes, new staff hires, and student crises.

**TABLE 2:** Recruitment and training of youth co‐researchers.

Cohort group	Cohort 1	Cohort 2	Cohort 3
Dates	February 2020–April 2021	March 2021–May 2021	August 2021–November 2021
Method of participation	In‐person and remote	In‐person	In‐person
Method of recruitment	Staff referral, information meeting, trial sessions	Classroom presentations, flyer, interest meeting, staff referrals	Classroom presentations, staff referrals, trial sessions
Incentive	Volunteer hours	No incentive due to distance learning	Volunteer hours
Curriculum	Institute of community research's YPAR, YPAR Hub	YPAR Hub (focus group lesson)	Institute of Community Research's YPAR, YPAR Hub (Focus group lesson)
Research tasks	Learned various research methodologies, selected research methods, designed student‐designed survey, outlined dissemination of student‐designed survey	Discussed student‐designed survey data, overview of focus group methods, decided areas of emphasis for school‐wide focus group, began developing focus group questions	Reviewed student‐designed data, overview of focus group methods, decided areas of emphasis for focus group, discussed focus group implementation
Number of youth co‐researchers	6	4–7	4–7

As explained above, cohorts were essential in ensuring continuity of research efforts developed by the YPAR team. The cohorts provided meaningful input throughout the process and took part in discussions and planning. The team engaged in critical reflection, proposed solutions, and acted with urgency for change.

### 3.2 Student‐designed survey findings

A total of 72 (66%) students responded to the survey. A summary of key findings is reported in Table 3. Nearly three‐quarters of student respondents reported taking care of younger family members, and close to one‐third shared that they feel very unsafe getting to and from school. Substance use was highest for marijuana compared to alcohol and other drugs (e.g., opiates) within the past 30 days. One‐quarter of students reported using marijuana in that timeframe, with 11% reporting use nearly every day (21–30 days). Using the recommended cut‐offs for the PHQ‐2 and GAD‐2 respectively, 20.8% of student respondents at the CHS met the screening criteria for Major Depressive Disorder (MDD) and 30.5% met anxiety‐screening criteria for a potential Generalized Anxiety Disorder (GAD) diagnosis if further evaluation were to be sought out.^26^

**TABLE 3:** Summary of student‐designed survey results.

Please check ALL of the following that you are responsible for	*n*		%			
Take care of younger siblings/ family members	52		72			
Taking people to doctor appointments (that you are not hired for)	7		10			
Working late, followed by waking up early to go to school	17		24			
Care taking for elder family members (grandparents, parents, etc.)	10		14			
Other	3		4			

During the past 30 days on how many days did you… (Please put an “X” in each box that applies to you)	0 days	1 day	2 days	3–9 days	10–20 days	21–31 days
EHave at least one drink of alcohol?	82%	8%	7%	1%	1%	0%
Use marijuana (smoke, vape, eat, or drink)?	75%	3%	4%	1%	6%	11%
Use any other drug, pill, or medicine for the purpose of getting high?	94%	0%	0%	1%	1%	3%

Have you ever chosen not to attend school, or chosen to leave school early or arrive to school late because you were under the influence?	Yes		No			
	12%		88%			

How safe do you feel getting to and from school (walking, taking the bus, etc.)?	Very safe	Safe	Neither safe/unsafe		Unsafe	Very unsafe
	3%	63%	19%		4%	31%
Percent of students who met screening criteria for Major Depressive Disorder via PHQ‐2	20.8%					
Percent of students who met screening criteria for Generalized Anxiety Disorder via GAD‐2	30.5%					

How many hours of sleep are you typically getting each night during the week?	1–3 h	4–6 h	6–8 h		8–10 h	
	5.6%	34.7%	40.3%		19.4%	

One of the final questions on the survey was open‐ended and asked students to share their reasons for not attending school. Responses included: “I get really bad anxiety and I feel like something is on my chest making it hard for me to breathe and also makes me feel noxious,” “Sometimes my anxiety gets really high and I literally cannot for the life of me show up to school like that,” “sick, doctors appointment, family problems,” “Lack of motivation I guess,” and “Very little sleep, anxiety, etc.”

### 3.3 Other findings

Evidence from this study suggests that school systems can learn from including youth as key stakeholders in educational reform. A student shared their frustration about the school board's request for student input from other schools and reflected on which students' input was valued.

They shouldn't be asking those straight A students, who have privilege, about how the school is working for them or how it can be better. It works for them. If they want to know how to make schools better, they should ask us. Students here who have been pushed to the back of the class and end up at a CHS.

School‐level changes resulted from YPAR as well. In Fall 2021, the school received Model CHS recognition from the CDE. Achieving this award signals a dramatic change from the CSI eligible status that initially prompted the collaborative agreement that began this work. The YPAR research team was involved in providing letters of recommendation and interviews for the award. The implementation of YPAR was highlighted as an example of a comprehensive service for students who are “at‐risk.” Additional outcomes included the creation of intentional youth spaces in response to co‐researchers' expressed need for a room to unwind. In Fall 2021, the school converted a classroom into a room with couches and tables for students to use as needed.

Participants of YPAR engaged in several transformational experiences as a result of skills they developed during YPAR. Gathered from informal conversations and YPAR sessions with youth co‐researchers, student feedback often suggested that they valued the YPAR content (e.g., “We have intellectual conversations about *why* these things are happening”), their connection to other students (e.g., “to socialize and being homies with people I didn't expect”), and the group rapport (e.g., “This is a safe place for me to talk about stuff,” “It's not chaotic here”). In addition, several YPAR student co‐researchers were recommended and attended the California Association of Student Councils (CASC) as a leadership opportunity. One YPAR student presented at the 2020 National Association for School Psychologists conference. Several YPAR students were nominated for and supported to obtain local, state, and national scholarships. In addition, throughout the process, the graduate research assistant supported students with scholarship applications, provided crisis support, trained the CHS alumni to become mentors for current students, and spoke at a local school board meeting to advocate for the community image of the continuation school students.

## 4 DISCUSSION

Given the limited research of YPAR implementation within CHS, this study sought to explain the process of YPAR implementation within a CHS over three academic years and describe outcomes of the YPAR research. As a methodological approach, YPAR was crucial in creating a space for youth voices, honoring their lived experiences, and placing them as key stakeholders when conducting and implementing research. YPAR was also a critical tool in acknowledging the systemic concerns that contributed to students' positionality and their experience with the US educational system. The implementation of this project was intentional about discussions of the school system failing them and holding conversations with a CRT lens about social injustices. As one student noted, “I found this stuff important. I wasn't even thinking about beforehand and then it was important to me.” Notably, this study was also conducted in the context of the COVID‐19 pandemic and distance learning, which impacted the implementation, advocacy efforts, and results of the study.

### 4.1 YPAR implementation in CHS

This project has several important implications for YPAR implementation within CHSs. First, CHS students are highly mobile between their traditional high school and the CHS, which can affect group rapport, continuity of group norms, selected methodology, and ownership of the YPAR project. With a cohort model of implementation, it is likely that adult researchers will need to negotiate and reconcile previous youth co‐researchers' requests with new youth co‐researchers. Thus, we found it beneficial to allow youth co‐researchers from previous cohorts to attend the first YPAR sessions of the new cohort to integrate knowledge and experiences. While implementing YPAR within CHSs, adult researchers should remain flexible and adaptable to the new youth co‐researchers' needs and requests.

### 4.2 Unintended outcomes of YPAR in CHS

This study also illuminated unintended outcomes of YPAR implementation. Supporting students with scholarship applications, college and employment applications, and crisis intervention were all frequent occurrences. Although the CHS often had resources readily available for students to access in these areas, youth co‐researchers often sought out the adult researcher's input and guidance. We found that it was necessary to frequently disclose limitations to confidentiality and have resources on hand to direct students when necessary.

### 4.3 YPAR research findings

Descriptive data from the student‐designed survey provided rich data about barriers to attendance. The majority of student respondents (72%) endorsed missing school to support family members such as taking care of siblings, accompanying family members to appointments, or supporting parents struggling with substance use disorders. Previous research has underscored that students who attend alternative education programs often face challenges that their traditional high school peers may not, for example potential involvement in the foster care system and experiencing lower socioeconomic status.^8^ This study adds to the literature about potential challenges CHS may experience including additional responsibilities CHS students may have that are not consistent with traditional high school peers.

One of the main goals for student researchers was to better understand their peers' mental health concerns. In the present study, 30.5% of student respondents met screening‐criteria for GAD, similar to other research studies that also used the GAD‐2 as a screening tool for adolescents.^30^ Results for the PHQ‐2, which is intended to screen for MDD, revealed that 20.8% of the student respondents met screening criteria. In a comparable study sample, researchers found that 7.4% of their sample met screening criteria on the PHQ‐2.^31^ While it is possible that screening rates for depression were higher in our study sample because students were screened during the pandemic and most previous studies were conducted before COVID‐19, the rates are nonetheless high. Notably, youth were not diagnosed with MDD or GAD, but rather the results from these screeners highlighted the likelihood of CHS students being diagnosed with MDD or GAD if they sought further evaluation. During YPAR discussions, students mentioned that some of their peers “just didn't feel like going” due to feelings of general sadness. They also mentioned that students sometimes miss school due to health concerns that have not been addressed, sometimes due to a lack of insurance. In other cases, their peers have lied to their guardians about having physical symptoms (e.g., stomach aches, headaches) so that they would be allowed to miss school due to underlying emotional distress. Physical symptoms may indicate distress; a systematic review of 53 studies that explored reasons why youth do not access or seek mental health support revealed that perceived public/social stigma was the second most common (92%) theme among youth respondents behind limited mental health knowledge.^32^

### 4.4 Future directions and implications

Evidence from this study suggests that school systems can learn from including youth voices as key stakeholders in educational reform. Specifically, CHS are in a unique position to support students who have been failed by the school system and therefore should be at the forefront of educational reform. This research is consistent with literature that emphasizes that “vulnerable” youth are crucial and should be providing input for shaping and reforming educational policy and practice.^22^, ^33^, ^34^ Related to our research findings, we recommend that CHS engage in youth‐led mental health screening that includes measures of somatization and stigma to guide multi‐tiered interventions that address the unique experiences of CHS students and their mental health. CHS would also benefit from developing programs for families in consideration of their students' family responsibilities.

### 4.5 Limitations

Although this project was intended to highlight the process of YPAR within a specific CHS, there are limitations and challenges the authors found notable. Generalizability is limited given that this study was conducted in California and state education laws differ, namely compulsory education that largely affects the study participants and youth co‐researchers. In addition, demographics of this CHS may not be representative of other CHS or alternative education systems and therefore, there may be additional considerations in a different social context. Youth co‐researchers were students who were identified as “at‐risk” of not graduating at 16 years of age, which in some states, would allow them to cease their public education if they chose. Additionally, findings from the student‐designed survey were limited to questions considered and asked by the graduate student researcher and youth co‐researchers, so were unable to identify other reasons for disengagement.

## 5 CONCLUSION

This project contributes to the limited literature of YPAR implementation within CHS, offering an example of YPAR implementation across three academic years with highly mobile youth, and provides insight into barriers to school attendance. Student co‐researchers demonstrated the critical need to engage youth who have historically been failed by the US education system in order to implement educational reform that serves all students.

## FUNDING INFORMATION

This project was funded by a grant from the California Department of Education from the Comprehensive Support and Improvement Funding. The first author is a recipient of a fellowship from the Robert Wood Johnson Foundation Health Policy Research Scholars Program.

## Supporting information


**Appendix S1.** Supporting information
