# Supplementary material for: Implementing youth participatory action research at a continuation high school
Source: Health Serv Res. 2023 Jun 6;58(Suppl 2):198–206. doi: 10.1111/1475-6773.14190 (PMC10339165; doi:10.1111/1475-6773.14190)
Supplement: Supplementary file 1 — Appendix S1. Supporting information [file HESR-58-198-s001.docx]

Appendix A

1. How long have you been enrolled at XXX?

- Less than 1 quarter
- 1 Quarter
- 2 Quarters
- More than 2 quarters
- 1 Year
- Write your own answer: ________________________________________

2. How often has quarantine affected your motivation for schoolwork?

- Not at all
- Sometimes
- Most of the time
- Almost always

3.Please check ***All*** of the following that you are responsible for:

|  | Take care of younger siblings/ family members |
| --- | --- |
|  | Taking people to doctor appointments (that you are not hired for) |
|  | Working late, followed by waking up early to go to school |
|  | Care taking for elder family members (grandparents, parents, etc.) |
|  | Other (please explain): _________________________________________ |

4. During the past 30 days on how many days did you… (Please put an “X” in each box that applies to you)

|  | 0 days | 1 day | 2 days | 3-9 days | 10 – 19 days | 20 – 30 days |
| --- | --- | --- | --- | --- | --- | --- |
| Have at least one **drink of alcohol?** |  |  |  |  |  |  |
| Use **marijuana** (smoke, vape, eat, or drink)? |  |  |  |  |  |  |
| Use any **other drug, pill, or medicine** for the purpose of getting high**?** |  |  |  |  |  |  |

5. Thinking back to last school year (2019-2020), before the school closure, how often did you attend school under the influence?

- Never
- A couple times in the past year
- A few times a week
- Every day of the week

6. During this school year (2020-2021), during Distance Learning, how often have you attended your Zoom classes under the influence?

- Never
- A couple times in the past year
- A few times a week
- Every day of the week

7. Have you ever chosen not to attend school at all or chosen to leave school early or arrive late to school because you are under the influence?

- Yes
- No

8a. Do you rely on the bus system get to school?

- Yes
- No

8b. In your experience, when you rely on taking the bus to school (this year, or in past school years), how many days per week is the bus late?

- - 1 – 2 days
  - 3 – 4 days
  - Usually not late

9. How safe do you feel getting to and from school (walking, taking the bus, etc.)?

- Very safe
- Safe
- Neither safe nor unsafe
- Unsafe
- Very unsafe
- If responded unsafe or very unsafe – please explain: ________________________________________________________________________________________________________________________________________________

10. Over the past ***2 weeks***, how often have you been bothered by any of the following problems?

|  | **Not at all** | **Several days** | **More than half the days** | **Nearly every day** |
| --- | --- | --- | --- | --- |
| Little interest or pleasure in doing things? | 0 | 1 | 2 | 3 |
| Feeling down, depressed or hopeless | 0 | 1 | 2 | 3 |

11. Over the past ***2 weeks****,* how often have you been bothered by any of the following problems?

|  | **Not at all** | **Several days** | **More than half the days** | **Nearly every day** |
| --- | --- | --- | --- | --- |
| Feeling nervous, anxious, or on the edge? | 0 | 1 | 2 | 3 |
| Not being able to stop or control worrying? | 0 | 1 | 2 | 3 |

12. Have any of the feelings (from the 4 previous questions) affected your attendance at school?

- Yes
- No

13. Do you have any health-related concerns that has affected your academic work or attendance? (example: seizures, untreated poor eyesight, untreated health concerns, constant doctor appointments)

- Yes
- No

14. How many hours of sleep are you typically getting each night during the week?

- 1 – 3 hours
- 4 – 6 hours
- 6 – 8 hours
- 8 – 10 hours
- More than 10 hours

15. What are you some reasons you are not getting enough sleep? (sharing a room with others, phone, watching TV, can’t sleep, stress/anxiety) ***Please Explain Below.***

____________________________________________________________________________________________________________________________________________________________

15. What are some other reasons you might ***NOT*** be attending school?

______________________________________________________________________________

______________________________________________________________________________

16. Do you know anyone in a suspicious relationship where your friend might be in danger?

- Yes
- No

17. Have you seen anyone you know with random expensive gifts? (Name-brand belts/clothes, nice purses, expensive jewelry, laptops, phones, etc.)

- Yes
- No

18. What motivates you to attend school, other than graduating? Please check ***ANY*** of the following (can select multiple)

|  | A sense of community |
| --- | --- |
|  | Having adults to talk to |
|  | Food source (breakfast, lunch, etc.) |
|  | A safe place to be |
|  | Your favorite class |
|  | Other (please explain):_______________________________________________________ |

19. Is there anything else you would like to tell us about your experience at XXX?

____________________________________________________________________________________________________________________________________________________________

**Thank you for taking the time to complete this survey and helping us to improve our school and our resources for you and your classmates.**

If you are in need of additional support, please talk to any trusted adult in our school community to get access to a variety of other resources. Here are some helpful emails and phone numbers.

[resources masked for privacy]
